# Superwide Left Atrial Circumferential Ablation for Persistent Atrial Fibrillation: A Randomized Controlled Trial

**DOI:** 10.1111/jce.16700

**Published:** 2025-04-30

**Authors:** Yue Cao, Wei Du, Yalan Fei, Hao Yang, Meng Wang, Qingshan Dong, Xianjin Li, Shijie Li, Bing Han

**Affiliations:** ^1^ Xuzhou Clinical School of Xuzhou Medical University Xuzhou China; ^2^ Department of Cardiology Xuzhou Central Hospital Xuzhou China

**Keywords:** arrhythmia recurrence, persistent atrial fibrillation, pulmonary vein antrum isolation, randomized controlled study, superwide left atrial circumferential ablation

## Abstract

**Background:**

It remains to be answered whether further enlargement of the isolation area in the left atrium (LA) could produce better effectiveness than pulmonary vein antrum isolation (PVAI) for treatment of atrial fibrillation (AF).

**Aims:**

To assess the effectiveness of superwide left atrial circumferential ablation (SWLACA) for treatment of persistent AF (PeAF) through a randomized controlled study.

**Methods:**

A total of 248 patients (male 63.7%, median age 65 years) with PeAF were randomly divided into the SWLACA group and PVAI group. The circumferential lines were located 2–3 cm from the PV ostia in the SWLACA group and approximately 1 cm in the PVAI group, respectively. The primary endpoint was the recurrence of atrial tachyarrhythmia after a single procedure.

**Results:**

Conduction block between LA and pulmonary veins (PVs) were achieved in all patients. The isolation areas were obviously larger in the SWLACA group (*p* < 0.001). Compared with the PVAI group, the SWLACA group was associated with a longer procedure duration (*p* = 0.013) and fluoroscopic time (*p* = 0.038). During the 12‐month follow‐up, the overall recurrence rate of atrial tachyarrhythmia after a single procedure was not significantly different between the two groups (21.0% vs 26.6%; *p* = 0.297). However, the SWLACA group had significantly fewer AF recurrences (12.9% vs. 25.0%; *p* = 0.015), and more atrial tachycardia recurrences (8.1% vs. 1.6%; *p* = 0.018). After multiple procedures, the SWLACA group had a significantly higher total arrhythmia recurrence‐free rate (*p* = 0.030).

**Conclusions:**

Compared with PVAI, although SWLACA did not significantly decrease the overall arrhythmia recurrence rate for PeAF, it was associated with a notable reduction in the recurrence of AF.

## 1 Introduction

Catheter Ablation has become an essential therapy for atrial fibrillation (AF), with better efficacy in maintaining sinus rhythm, improving symptoms, and enhancing the quality of life compared to medical therapy [1, 2]. The electrical isolation of the pulmonary veins (PV) is widely considered as the cornerstone ablation strategy [3, 4]. Previous studies have displayed that the recurrence of atrial tachyarrhythmia after pulmonary vein antrum isolation (PVAI) is less likely than that after segmental ostial ablation, especially for persistent AF (PeAF) [5, 6, 7, 8, 9]. However, it has yet to be answered whether further enlargement of the isolation area could bring more benefit to AF ablation. According to a retrospective study, a larger isolation area was associated with a significantly lower recurrence rate [10]. In the past few years, our center has been attempting to perform superwide left atrial circumferential ablation (SWLACA), which involves creating circumferential ablation lines 2–3 cm away from the PV ostia. The feasibility and safety are confirmed through our initial experience. We plan to conduct a randomized controlled study to compare the effectiveness of SWLACA and PVAI for the treatment of PeAF.

## 2 Methods

### 2.1 Study Design and Population

Patients with PeAF who underwent their first catheter ablation at Xuzhou Central Hospital between January 2019 and October 2021 were enrolled in this single‐center, prospective, randomized controlled study. To be eligible for this study, participants had to meet the following criteria:(1) age between 18 and 80 years; (2) diagnosed with PeAF based on their medical history and ECG/Holter recordings; (3) ineffective or intolerant response to antiarrhythmic drugs or declining long‐term drug therapy; (4) agreeing to join this study and signing an informed consent form. The exclusion criteria were as follows: (1) moderate to severe valvular disease; (2) congenital heart disease; (3) history of previous catheter ablation for AF; (4) left atrial (LA) diameter of 60 mm or greater; (5) left ventricular ejection fraction less than 40%; (6) significant pulmonary dysfunction; (7) thrombus in the LA; (8) contraindication to anticoagulation; (9) history of thoracotomy or cardiac surgery; (10) pregnant women; (11) expected life expectancy of fewer than 12 months; (12) unable to provide informed written consent. The study has been authorized by the Medical Ethics Committee of Xuzhou Central Hospital (XZXY‐LJ‐20180620‐008) and registered on the Chinese Clinical Trial Registry (ChiCTR1900020764). After written informed consents were signed, the enrolled patients were randomly divided into the SWLACA and PVAI groups.

### 2.2 Catheter Ablation Procedure

All patients should take oral anticoagulants for at least 1 month and undergo transesophageal echocardiography or computed tomography (CT) to exclude LA thrombus within 24 h before the procedure. Antiarrhythmic drugs, except amiodarone, were discontinued for at least five half‐lives.

**Figure 1:**
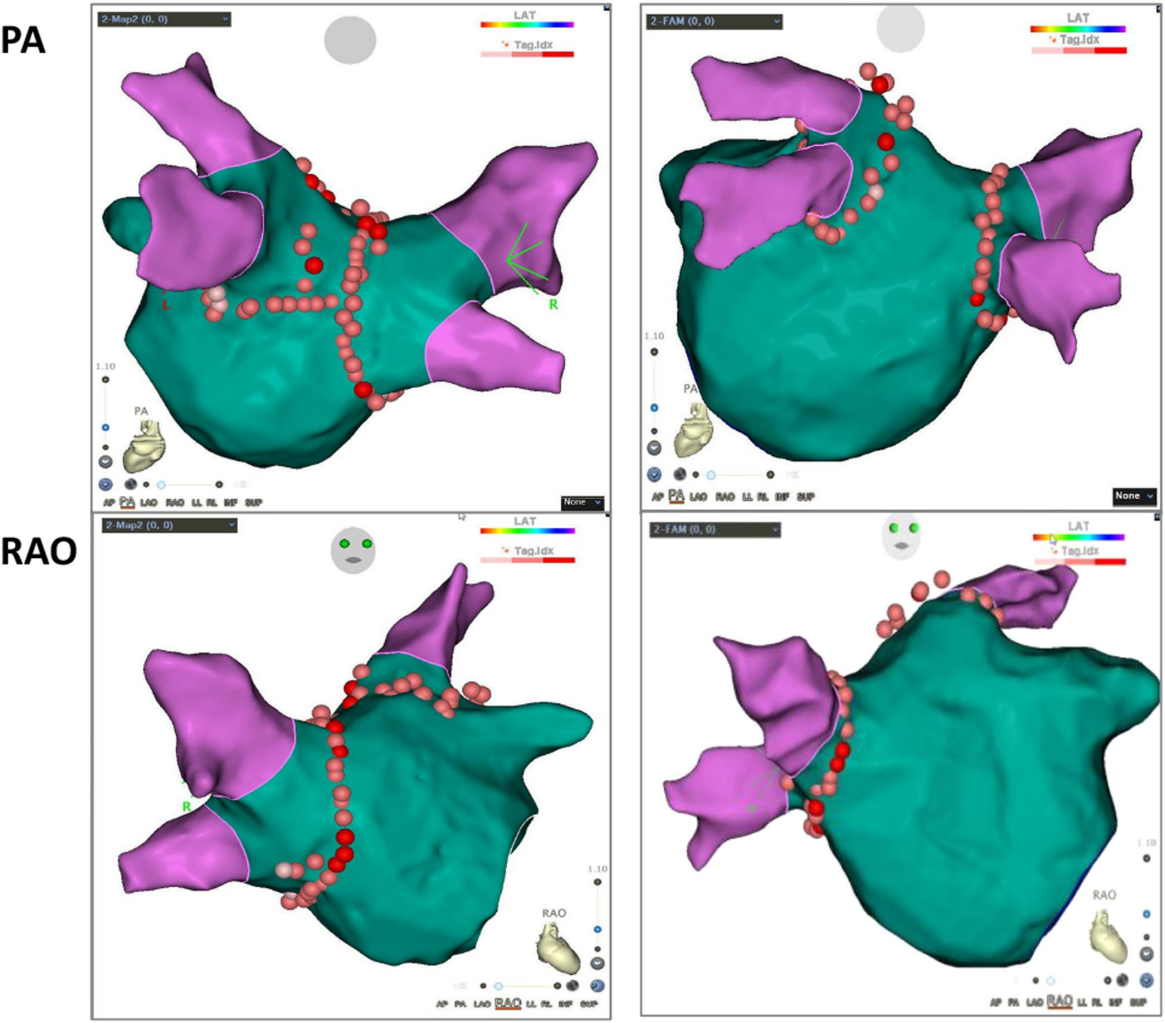
CARTO Images of SWLACA and PVAI. The images of SWLACA (shown in left column) displayed a single ablation line shared by the right and left side rings on the posterior walls. In comparison to the images of PVAI (shown in the right column), it was also observed that a larger area of the anterior wall adjacent to the right PV was enclosed within the isolation circle. PA = posteroanterior, PVAI = pulmonary vein antrum isolation, RAO = right anterior oblique, SWLACA = super wide left atrial circumferential ablation.

The procedures were performed under local anesthesia combined with intravenous morphine. After double transseptal punctures, two 8.5 F SL1 sheaths (St. Jude Medical, St. Paul, MN) were advanced into the LA. Systemic anticoagulation was achieved by intravenous administration of heparin, maintaining an activated clotting time (ACT) of 300–350 s throughout the whole procedure. A circular mapping catheter (Lasso, Biosense‐Webster, Diamond Bar, CA) was introduced into the LA through a transseptal sheath to record PV potentials. An irrigated contact force–sensing catheter (ThermoCool SmartTouch or ThermoCool SmartTouch SF, Biosense Webster, Diamond Bar, CA) was used for mapping and ablation. The ablation procedure was performed under the guidance of an electro‐anatomic mapping system (CARTO 3, Biosense Webster, Irvine, CA). In all patients, circumferential ablation was first performed around the right‐side PV and then the left‐side. In the SWLACA group, the circumferential ablation lines were positioned 2–3 cm away from the PV ostia, except at the anterior LA‐PV junction, which was bounded by the ridge between the PV and the left atrial appendage (Figure 1). In contrast, in the PVAI group, they were positioned at a closer distance of approximately 1 cm from the ostia (Figure 1). In both groups, the distances between the ablation lines and the PV ostia were identified by selective PV angiography combined with 3D electroanatomical mapping. It was important to note that for patients in the SWLACA group, the transseptal puncture needed to be performed at the anterior‐inferior part of the atrial septum to conveniently complete the large‐area isolation of the LA anterior wall next to the right PV antrum. The site for the puncture was determined according to the position of the CS electrode, the contour of the left atrial posterior wall, and the image of other anatomical structures, such as the thoracic vertebrae, viewed from the right anterior oblique angle projection. If there were challenges in positioning, a left atrial angiography through the long sheath after completing the first puncture was performed following the initial puncture to help accurately identify the site for the second puncture. The isolation area was calculated by the CARTO electro‐anatomic mapping system. Radiofrequency energy was delivered with a maximum power of 30 W on the posterior wall and 35 W on the anterior wall. The contact force was limited to a maximum of 20 for each RF discharge, particularly at the posterior wall, to minimize the risk of esophageal damage. The target ablation index (AI) values were set between 380 and 400 for the posterior wall and between 420 and 450 for the anterior wall and roof segment. The distance between adjacent ablation lesions was required to be less than 4 mm. The temperature of the radiofrequency energy catheter was limited to 43°C, with irrigation rates of 17–30 mL/min. If AF persisted after complete electrical isolation of all PVs, sinus rhythm (SR) was restored by direct current (DC) cardioversion. The conduction block between the LA and PV was confirmed by the disappearance of PV potential or the appearance of spontaneous potential. If AF converted to regular atrial tachycardia (AT) during ablation, the LA and RA activation map was conducted. Only when cavotricuspid isthmus (CTI) dependent atrial flutter was identified, ablation along the CTI was performed, otherwise, AT was terminated by DC cardioversion. Ablation of non‐PV triggers was based on the presence or absence of spontaneous AF, AT or frequent atrial ectopic beats.

### 2.3 Postprocedural Management and Follow‐Up

All patients were required to take novel oral anticoagulants (NOACs) or warfarin. The antiarrhythmic drugs (amiodarone, propafenone or dronedarone) were used during the 3‐month blanking period unless there existed bradyarrhythmia. Regular telephone and clinic follow‐ups were provided for all patients. 7‐day Holter monitoring was performed at 3, 6, and 12 months postoperatively. Any atrial tachyarrhythmias lasting more than 30 s beyond the 3‐month blanking period was considered a recurrence. In case of recurrence, the treatment options were based on the patient's preference.

### 2.4 Study Endpoints

The primary endpoint was the first documented atrial tachyarrhythmias recurrence by electrocardiography or implanted device after a single procedure during a 12‐month follow‐up period. The secondary endpoints included: the incidence of severe intra and postoperative complications, procedure duration, fluoroscopy time, recurrence of different types of atrial tachyarrhythmias.

### 2.5 Statistical Analysis

Normally distributed continuous variables were expressed as mean ± standard deviation (SD) and compared by the Student *t*‐test; non‐normally distributed data were presented as the median with interquartile range (IQR) and compared by the Mann‐Whitney U test. Categorical variables were described in numbers or percentages and compared through the χ^2^ test. Arrhythmia‐free survival was estimated using Kaplan‐Meier curves. Log‐rank test was used to compare survival differences between different groups of patients. A two‐sided *p* < 0.05 was considered statistically significant. Statistical data analysis was performed with SPSS 26.0 software (IBM Co, Armonk, NY, USA).

## 3 Results

### 3.1 Patient Characteristics

A total of 248 patients were enrolled in this study, with a median age of 65 years (IQR: 56–70 years). Of those patients, 158 (63.7%) were male, and 89 (35.9%) had long‐standing PeAF (LSPAF). 124 patients were assigned into the SWLACA group and the other 124 into the PVAI group. Both groups had similar baseline characteristics (Table 1).

**Table 1:** Baseline patient characteristics.

	Total (*n* = 248)	SWLACA (*n* = 124)	PVAI (*n* = 124)	*p* value
Age (years)	65.0 (56.3, 69.8)	67.0 (57.0,70.0)	62.5 (56.0, 69.0)	0.086
Male sex	158 (63.7)	78 (62.9)	80 (64.5)	0.792
BMI (kg/m^2^)	25.8 ± 2.9	25.5 ± 2.9	26.1 ± 3.0	0.125
Duration of AF (months)	4.5 (1.0, 24.0)	3.0 (1.0, 28.0)	6.0 (1.0, 22.3)	0.581
LSPAF	89 (35.9)	42 (33.9)	47 (37.9)	0.508
Previous history				
Hypertension	121 (48.8)	58 (46.8)	63 (50.8)	0.525
Coronary heart disease	45 (18.1)	22 (17.7)	23 (18.5)	0.869
Diabetes mellitus	29 (11.7)	11 (8.9)	18 (14.5)	0.167
Stroke/TIA	42 (16.9)	22 (17.7)	20 (16.1)	0.735
COPD	10 (4.0)	5.0 (4.0)	5.0 (4.0)	1
Congestive heart failure	30 (12.1)	16 (12.9)	14 (11.3)	0.697
CHA2DS2‐VASc score	2 (1.3)	2 (1.3)	2 (1.3)	0.476
HAS‐BLED score	1 (0.2)	1 (1.2)	1 (0.2)	0.238
LA diameter (mm)	40.0 (38.0, 44.0)	40.0 (38.0, 44.0)	40.0 (38.0, 44.8)	0.374
LVEF (%)	56 (55, 58)	55 (54, 58)	56 (55, 58)	0.051

*Note:* Values are presented as *n* (%), as mean ± SD or median (P25, P75).

Abbreviations: AF, atrial fibrillation; BMI, body mass index; COPD, chronic obstructive pulmonary disease; LA, left atrium; LSPAF, long‐standing persistent atrial fibrillation; LVEF, left ventricular ejection fraction; PVAI, pulmonary vein antrum isolation; SWLACA, super wide left atrial circumferential ablation; TIA, transient ischemic attack.

### 3.2 Procedural Findings

The electrical conduction blocks between the LA and PVs were achieved in all patients. In the SWLACA group, the rates of first‐pass isolation for the right and left PV were 66.9% and 61.3%, respectively. These rates were significantly lower than those in the PVAI group, with 87.9% for the right PV and 84.7% for the left PV (*p* < 0.001). The SWLACA group had larger isolation areas around both left and right PVs than the PVAI group (left side: 18.0 [15.0, 20.6] cm^2^ vs. 12.5 [10.0, 15.1] cm^2^; right side: 15.5 [12.8, 18.0] cm^2^ vs. 12.0 [9.7, 14.0] cm^2^; *p* < 0.001). There were 117 patients in the SWLACA group who had a single ablation line shared by the right and left side rings on the posterior walls, but none in the PVAI group. Compared to the PVAI group, the isolation time of both the left and right PVs was longer in the SWLACA group (left side: 17.0 [12.0, 23.8] min vs 14.5 [10.3, 19.8] min; right side: 17.0 [13.0, 25.0] min vs 12.0 [10.0, 16.0] min; *p* < 0.01). The procedure duration (160.0 [140.0, 180.0] min vs 150.0 [120.0, 180.0] min; *p* = 0.013) and fluoroscopic time (19.4 [15.0, 24.0] min vs 17.0 [13.3, 22.6] min; *p* = 0.038) were also significantly prolonged in the SWLACA group (Table 2).

**Table 2:** Procedural findings during the initial ablation.

	Total (*n* = 248)	SWLACA (*n* = 124)	PVAI (*n* = 124)	*p* value
LAV (cm^3^)	150.8 (130.1, 174.1)	148.5 (131.0, 174.1)	152.0 (129.2, 174.1)	0.888
LASA (cm^2^)				
Pre‐PVI	131.0 (118.8, 150.0)	130.9 (120.0, 150.0)	131.5 (116.3, 149.7)	0.816
Post‐PVI	110.0 (97.8, 124.9)	101.1 (92.3, 114.9)	119.6 (105.8, 132.2)	< 0.001
Post‐/Pre‐PVI	85.8 (77.6, 91.8)	78.0 (73.1, 82.7)	91.4 (88.4, 93.7)	< 0.001
Additional ablation				
CTI dependent atrial flutter	6 (2.4)	2 (1.6)	4 (3.2)	0.679
Non‐PV triggers	18 (7.3)	11 (8.9)	7 (5.6)	0.328
Isolation area (cm^2^)				
Left side	15.1 (11.4, 18.5)	18.0 (15.0, 20.6)	12.5 (10.0, 15.1)	< 0.001
Right side	13.7 (11.2,17.8)	15.5 (12.8, 18.0)	12.0 (9.7, 14.0)	< 0.001
Ablation time (min)				
Left	16.0 (12.0, 22.0)	17.0 (12.0, 23.8)	14.5 (10.3, 19.8)	0.004
Right	15.0 (11.0, 20.0)	17.0 (13.0, 25.0)	12.0 (10.0, 16.0)	< 0.001
First pass isolation				
Right PV	192 (77.4)	83 (66.9)	109 (87.9)	< 0.001
Left PV	181 (73.0)	76 (61.3)	105 (84.7)	< 0.001
Procedural time (min)	150.0 (130.0, 180.0)	160.0 (140.0, 180.0)	150.0 (120.0, 180.0)	0.013
Fluoroscopic time (min)	18.0 (14.3, 23.0)	19.4 (15.0,24.0)	17.0 (13.3, 22.6)	0.038

Abbreviations: CTI, cavotricuspid isthmus; LASA, left atrial surface area; LAV, left atrial volume; PV, pulmonary vein; PVAI, pulmonary vein antrum isolation; PVI, pulmonary vein isolation; SWLACA, super wide left atrial circumferential ablation.

### 3.3 Follow‐Up and Endpoints

The scheduled follow‐ups were completed in all patients except one in the SWLACA group, who died of acute intracerebral hemorrhage 3 months after the first procedure. Another patient in the PVAI group suffered from cerebral embolism leading to hemiplegia during the periprocedural period. No other major complications occurred in both groups. Early recurrences within the 3‐month blanking period were less common in the SWLACA group (27 [21.8%] vs. 45 [36.3%]; *p* = 0.012).

**Figure 2:**
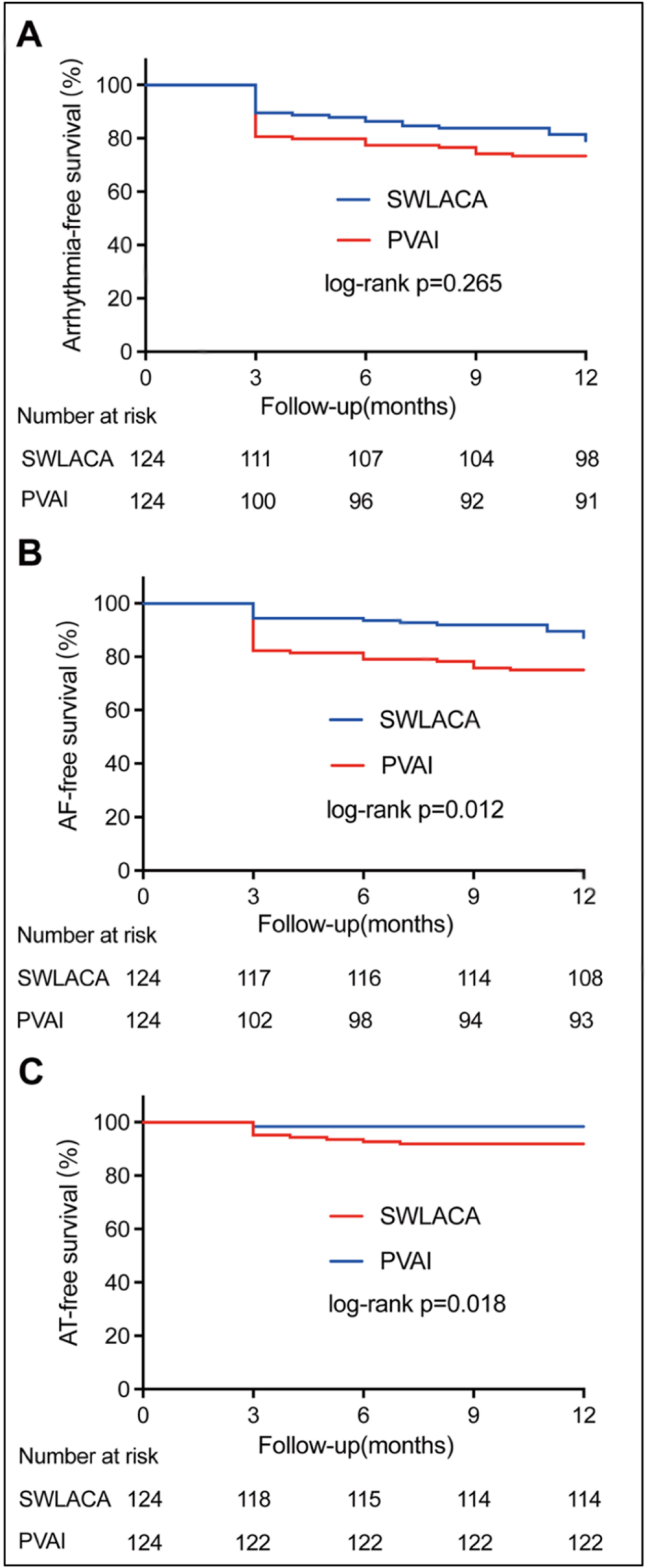
Kaplan–Meier curves showing time‐related freedom from arrhythmia (A), AF (B) and AT (C) after a single ablation procedure in SWLACA and PVAI groups. AF = atrial fibrillation, AT = atrial tachycardia, PVAI = pulmonary vein antrum isolation, SWLACA = super wide left atrial circumferential ablation.

During the 12‐month follow‐ups, a total of 59 (23.8%) patients had documented arrhythmia recurrence after a single procedure. Among those, 26 (21.0%) belonged to the SWLACA group and 33 (26.6%) to the PVAI group. The Kaplan–Meier analysis revealed no apparent differences in total arrhythmia‐free survival rates between the two groups (*p* = 0.265). However, the SWLACA group experienced significantly fewer recurrences of AF (16 [12.9%] vs. 31 [25.0%]; *p* = 0.015) and more recurrences of AT (10 [8.1%] vs. 2 [1.6%]; *p* = 0.018) compared to the PVAI group (Figure 2).

There were 12 (9.7%) and 11 (8.9%) patients receiving repeat catheter ablations due to recurrences in the SWLACA and the PVAI groups, respectively (*p* = 0.827). During the redo procedures, seven patients (70.0%) in the SWLACA group and 9 patients (75.0%) in the PVAI group exhibited at least one PV conduction recovery (*p* = 0.999). Mitral isthmus‐dependent ATs were demonstrated in 3 patients from the SWLACA group but none from the PVAI group. Following redo procedures, 1 patient in the SWLACA group and seven patients in the PVAI group experienced recurrences again. After multiple procedures, the SWLACA group had a significantly higher total arrhythmia recurrence‐free rate (*p* = 0.030) (Table 3).

**Table 3:** Follow‐up outcomes.

	Total (*n* = 248)	SWLACA (*n* = 124)	PVAI (*n* = 124)	*p* value
Antiarrhythmic drugs during the 3‐month blanking period	228 (91.9)	115 (92.7)	113 (91.1)	0.641
Early recurrence	72 (29.0)	27 (21.8)	45 (36.3)	0.012
Recurrence after single procedure	59 (23.8)	26 (21.0)	33 (26.6)	0.297
Recurrence as AF	47 (19.0)	16 (12.9)	31 (25.0)	0.015
Recurrence as AT	12 (4.8)	10 (8.1)	2 (1.6)	0.018
Receiving repeat ablation	23 (9.3)	12 (9.7)	11 (8.9)	0.827
Recurrence after multiple procedures	44 (17.7)	15 (12.1)	29 (23.4)	0.030

Abbreviations: AF, atrial fibrillation; AT, atrial tachycardia; PVAI, pulmonary vein antrum isolation; SWLACA, super wide left atrial circumferential ablation.

## 4 Discussion

The main findings of the current study were as follows: (1) The electrical conduction block between the LA and PVs could be achieved through SWLACA as successfully as through PVAI. (2) Compared to the conventional PVAI, SWLACA did not significantly reduce the arrhythmia recurrence for patients with PeAF after a single catheter ablation procedure. (3) More patients experienced recurrences of AT instead of AF with SWLACA.

It is widely confirmed that PVs play essential roles in the pathogenesis of AF, and therefore the strategies targeting the PVs have become the cornerstone for catheter ablation of AF [4]. In the initial years, focal or segmental ostial ablations were performed for the treatment of AF, with moderate effectiveness and relatively high incidence of PV stenosis [11, 12, 13]. Since the widespread use of three‐dimensional electroanatomical mapping, circumferential isolation around the PV antra with ablation line created approximately 1 cm from the ostia have become a more viable option to achieve the electrical conduction block between the LA and PVs [5, 6, 7, 8, 14]. Numerous studies have demonstrated that PVAI had a higher success rate and lower incidence of PV stenosis compared to segmental ostial ablation [15, 16]. However, whether further enlargement of the isolation area could lead to further improvement in therapeutic efficacy remains to be answered. One observational cohort study found that a larger isolation area was associated with a lower recurrence rate [10]. To investigate the feasibility and effectiveness of further expanding isolation areas around the pulmonary veins, we conducted this randomized controlled study. In our study, the circumferential ablation lines were positioned 2–3 cm away from the PV ostia for patients in the SWLACA group. In the majority of patients in this group, a single ablation line was shared by the right and left side rings on the posterior walls, actually achieving posterior wall isolation (PWI). While some non‐randomized studies indicated that combining PWI with PVI could reduce the recurrence rate following AF ablation [17, 18], two multicenter randomized trials, the POBI‐AF and CAPLA trials [19, 20], failed to identify any significant difference in efficacy between the PVI alone and the PVI + PWI strategies. Different from previous studies of the LA posterior wall isolation [17, 18, 19, 20], a larger area of the LA anterior wall next to the right PV was enclosed within the isolation circle in our study. To complete superwide isolation of the anterior wall, a key point needs to be mentioned: the transseptal puncture should be performed at the anterior‐inferior part of the atrial septum. Our results revealed that the electrical conduction blocks between the LA and PVs could be achieved in all patients of the SWLACA group as those of the PVAI group. However, in the SWLACA group, the first‐pass isolation rates for both the right and left PV were lower, while the ablation time and procedure duration were longer than those in the PVAI group.

During the 12‐month follow‐up, the total arrhythmia recurrence rates were not apparently different between the SWLACA and the PVAI groups after a single procedure. However, the SWLACA group had significantly fewer recurrences of AF and, conversely, more recurrences of AT than the PVAI group. Previous studies have revealed that AT and atrial flutter were more common after antral circumferential ablation than after ostial PV isolation [21]. Arentz et al. reported that a larger isolation area was associated with a lower overall arrhythmia recurrence rate and a higher incidence of AT [10]. Combined with the results of this study, it could be inferred that a wider isolation range surrounding PV might result in a lower incidence of AF recurrence but, to some extent, a higher incidence of AT recurrence. After multiple procedures, the SWLACA group had a significantly higher recurrence‐free rate than the PVAI group, which should be related to the difference in the proportion of recurrent arrhythmia types between the two groups after a single procedure. Some studies have confirmed that if patients experienced AT recurrences following AF ablation, the outcomes after repeat procedures would be significantly better than those with recurrent AF [22, 23]. It should be mentioned that in this study, less than half of the recurrent patients received repeat catheter ablations, so the results after multiple procedures should be interpreted cautiously.

## 5 Limitations

The main limitation of this study is that it was conducted in a single center. Moreover, it might require a larger sample size than initially estimated. Multicenter studies with larger sample sizes are needed to confirm our findings further. In addition, since only 7 days Holter monitoring was utilized to assess arrhythmia recurrences, the actual recurrence rate may have been underestimated. However, this situation was likely equivalent for both groups, so it should have little impact on the results. Lastly, since only patients with PeAF were included in this study, the results may not be applicable to those with paroxysmal AF.

## 6 Conclusion

Although SWLACA did not significantly decrease the overall recurrence rate of atrial arrhythmia after a single ablation procedure for PeAF, it did lead to a reduction in the recurrence of AF when compared to conventional PVAI, which revealed the potential advantages of superwide LA isolation. However, these findings require further confirmation through multicenter studies with larger sample sizes.

## Acknowledgments

This study received the support of the BiosenseWebster Inc (Investigator initiated study grant number: IIS‐498); the National Natural Science Foundation of China (grant number: 82370329); the Xuzhou Science and Technology Project(grant number: KC21155); the Xuzhou Medical leading Talents Project (grant number: XWRCHT20210032); the Project of Xuzhou Health Committee (grant number: Xuzhou Health Science and Education [2017] No.22).

## Ethics Statement

The study has been authorized by the Medical Ethics Committee of Xuzhou Central Hospital (XZXY‐LJ‐20180620‐008) and registered on the Chinese Clinical Trial Registry (ChiCTR1900020764). Informed consent was obtained from all individual participants included in the study. All authors have reviewed and agree with the content of the article for publication.

## Conflicts of Interest

The authors declare no conflicts of interest.

## Data Availability

The data underlying this article will be shared on reasonable request to the corresponding author.
